# The threshold of rule productivity in infants

**DOI:** 10.3389/fpsyg.2023.1251124

**Published:** 2023-10-30

**Authors:** Rushen Shi, Emeryse Emond

**Affiliations:** Université du Québec à Montréal, Montreal, QC, Canada

**Keywords:** rule learning, generalization, productivity, language, infant, implicit/incidental learning

## Abstract

Most learning theories agree that the productivity of a rule or a pattern relies on regular exemplars being dominant over exceptions; the threshold for productivity is, however, unclear; moreover, gradient productivity levels are assumed for different rules/patterns, regular or irregular. One theory by Yang, the Tolerance Principle (TP), specified a productivity threshold applicable to all rules, calculated by the numbers of total exemplars and exceptions of a rule; furthermore, rules are viewed as quantal, either productive or unproductive, with no gradient levels. We evaluated the threshold and gradience-quantalness questions by investigating infants’ generalization. In an implicit learning task, 14-month-olds heard exemplars of an artificial word-order rule and exceptions; their distributions were set closed to the TP-threshold (5.77) on both sides: 11 regular exemplars vs. 5 exceptions in Condition 1 (productiveness predicted), and 10 regular exemplars vs. 6 exceptions in Condition 2 (unproductiveness predicted). These predictions were pitted against those of the statistical majority threshold (50%), a common assumption which would predict generalization in both conditions (68.75, 62.5%). Infants were tested on the trained rule with new exemplars. Results revealed generalization in Condition 1, but not in Condition 2, supporting the TP-threshold, not the statistical majority threshold. Gradience-quantalness was assessed by combined analyses of Conditions 1-2 and previous experiments by Koulaguina and Shi. The training across the conditions contained gradually decreasing regular exemplars (100, 80, 68.75, 62.5, 50%) relative to exceptions. Results of test trials showed evidence for quantalness in infants (productive: 100, 80, 68.75%; unproductive: 62.5, 50%), with no gradient levels of productivity.

## 1. Introduction

Productive knowledge of a rule or a pattern enables one to apply the regularity to novel instances. Even young children show productive knowledge. For example, they give the plural form of an invented word “wugs” upon hearing the singular “a wug” (Berko, 1958), and they produce overgeneralizations such as “goed” (e.g., Pinker, 1995). Much attention has been devoted to the understanding of how rules/patterns are represented and how they are acquired.

Models of linguistic representations for rules/patterns differ in certain basic theoretical constructs. Morphological processes, for example, have been intensely debated. In some theories all morphological patterns are represented as abstract rules (e.g., Chomsky and Halle, 1968), regulars and irregulars. The dual-route model (e.g., Pinker, 1999) proposes the co-existent representations of abstract rules for regulars and memorized exemplars for irregulars. At the other extreme, no abstract symbolic rules are represented; rather, exemplars are encoded as clusters of constructions or as networks with shared features (e.g., Rumelhart and McClelland, 1986; Goldberg, 2016), and if they are sufficiently regular, productivity can emerge. According to the radical exemplar model of Ambridge (2020), generalizations occur in language use through analogy with stored (non-abstract) exemplars in the representation that have similar surface forms and semantic/contextual information. Across these models, a shared view is that regular and irregular representations are in competition to yield the predicted productivity of regular rules/patterns and overgeneralizations (or the avoidance of the latter). Although most of them focus on the nature of representations, the models offer insights that impact learning.

While rules can be learned through explicit teaching, as is often done in schools, most essentials of morphosyntactic regularities are acquired during preschool years from exemplars heard in natural environment without any instruction. The question then concerns how rules/patterns become productive for children. Drastically different theoretical positions have been proposed in the field. For some researchers, morphosyntactic structures are productive and abstract from the onset of acquisition, with no need for statistical learning from the distributional properties of the input. For example, Valian et al. (2009) claim that syntactic categories and structures are not only abstract, but also innate. Based on their analysis of the determiner-noun productions in CHILDES corpora, they concluded that young children demonstrate adult-like full productivity as soon as they start combining determiners and nouns. In contrast, other corpus studies (e.g., Pine and Lieven, 1997; Pine et al., 2013) reported that children’s initial NP productions are memorized exemplars, and that abstraction and productivity develop (through analogy-based inductive learning) in gradual stages. The mixed results and conclusions across studies reflect the methodological difficulties in working with natural speech samples, both for characterizing input distributions and for evaluating children’s productivity.

Various induction-based models attempted to specify the input properties enabling the learning of productive rules/patterns when regular and irregular exemplars are co-present. Type frequency, namely, the number of different exemplars of a rule/pattern, is important in most models (e.g., Plunkett and Marchman, 1991; Baayen, 1993; Bybee, 1995; Yang, 2016). The ways to quantify the contribution of type frequency differ across theories. In their connectionist model of morphological learning, Rumelhart and McClelland (1986) showed that input needed to contain a large number of regular exemplars (i.e., high type frequency^1^) relative to exceptions before a network start generalizing. Bybee (1995) suggested that to achieve productivity, regular exemplars must be dominant in type frequency relative to exceptions, although the exact level of the types was unspecified. Baayen’s model (Baayen, 1993) proposed indices aiming at comparing morphological processes (e.g., regulars vs. irregulars within a rule, or unrelated rules) in a language, by quantifying them into varying degrees of productivity. The variables in the calculation of his indices, which are properties of spoken corpora, seem relevant for learning. In particular, the productivity index p* = n/h divides the number of singletons *n* with a given suffix by the sum of singletons *h* with a range of suffixes in a corpus. For example, regular and irregular suffixes can be ranked in their p* values, with the higher type frequency of regular exemplars producing a higher p* (and thus more productive) than irregular exemplars. The emphasis on singletons (i.e., non-repeated exemplars) in p* highlights the importance of low token frequency for regular exemplars, an indication of generalization to novel instances, according to Baayen. Bybee’s learning model also stresses the importance of high type frequency and low token frequency of regular exemplars. Nearly all theories assume that rules/patterns vary in gradient degrees of productivity, and the threshold at which productivity emerges remains unspecified.

One theory (Yang, 2016), the Tolerance Principle (TP), departs from other learning theories in two major aspects. First, productivity cannot be gradient. The learner either has a productive rule or no rule. Second, TP specifies a productivity threshold, by *e* ≤ θ*_N_* where θ*_N_* =*N*/ln*N*, with *e* being the exceptions to the rule in question, θ*_N_* the TP-threshold, and N the sum of rule exemplars and the exceptions. Only type frequencies matter in this theory. Thus, if *e* does not exceed the threshold, the rule is learned and fully productive. If *e* is above the threshold, no rule is learned, and the learner resorts to rote memorization. Recent corpus studies showed support for TP-predicted (un)productiveness and quantalness of morphosyntactic patterns in adult speech (Pearl and Sprouse, 2021; van Tuijl and Coopmans, 2021; Henke, 2022) and in historical texts (Kodner, 2023). Furthermore, experiments on English-speaking 5-8-year-olds’ and Icelandic-speaking 2.5-to-6-year-olds’ production of morphological patterns (Schuler et al., 2016; Bjornsdottir, 2021) and on Russian-speaking 4-6-year-olds’ ordinal acquisition (de Vries et al., 2021) provided evidence for this theory. Yang (2016)’s analysis of the CHILDES corpora showed that children’s productivity and overgeneralization of English verb morphology was fully predictable by TP; notably, TP also correctly predicted the finding that children almost never produced irregularizations such as “wipe-wope” (only 0.02% such analogy errors in Xu and Pinker, 1995). Such errors would be predicted by analogy- and exemplar-based theories (e.g., Bybee, 1995; Ambridge, 2020).

Contrary to TP, gradient productivity was reported in many studies, typically using adults’ acceptance ratings or computer simulations of learning. Various linguistic rules/patterns were tested, such as English past tense (Albright and Hayes, 2003), final devoicing in morphological alternations in Dutch (Ernestus and Baayen, 2003), vowel harmony in Hungarian (Hayes et al., 2009), velar palatalization in Russian (Kapatsinski, 2010), and consonant cluster phonotactics in English (Olejarczuk and Kapatsinski, 2018). Participants were asked to produce novel words (i.e., the Wug test) or to make a perceptual response to novel word stimuli (e.g., force choice, parsing), and their performance showed gradient productivity, correlating with their ratings of the stimuli and matching the lexical distributions of the morphophonological patterns in the native languages.

Interestingly, these findings of gradient productivity for native language patterns resemble the performance of adults in artificial language learning experiments. Artificial language paradigms are advantageous because precise input characteristics during training can be specified and manipulated for determining learning mechanisms. A number of studies (Hudson Kam and Newport, 2005, 2009; Wonnacott et al., 2008; Austin et al., 2022) used a task in which the training input contained exemplars representing a phrase structure pattern, with certain levels of inconsistencies. Hudson Kam and Newport found that adults’ performance at test matched the inconsistent distribution of their training input (called “probability matching”), but children regularized, generalizing beyond the inconsistent input. Most notably, Austin and colleagues, who showed the same difference between adults and children in their recent study, further found that younger children (5- to 6-year-olds) regularized more than older children (7- to 8-year-olds). These results are important, demonstrating that adults and children have distinct learning mechanisms. While probability matching might be associated with adults’ gradient productivity, the regularization found in children, especially in younger ages, seems compatible with quantal representation.

Our interest thus concerns how infants at the earliest stage of cognitive development generalize, given inconsistent input. Do they probability match the input and show gradient productivity as do adults, or do they regularize as shown in 5- to 7-year-olds (Hudson Kam and Newport, 2005, 2009; Austin et al., 2022) and operate according to TP? In perceptual experiments that trained infants with 100% regular artificial language input, 7–9-month-old infants learned algebraic-like patterns (Marcus et al., 1999; Gerken, 2006), 12-month-olds learned grammatical categorization patterns (Gómez and Lakusta, 2004), and 14-month-olds generalized word-order movement (Koulaguina and Shi, 2013). Productive knowledge was demonstrated by infants’ discrimination of novel test exemplars that conformed with the trained pattern vs. violating it. Limited work exists on infants’ learning under inconsistent input. In Gomez and Lakusta infants who heard regular exemplars with 17% exceptions (by type frequency) succeeded in generalization, but infants exposed to 33% exceptions failed to show learning. In Koulaguina and Shi (2019), infants’ generalization of word-order patterns was successful when the training input contained 20% exceptions, but unsuccessful when exceptions were increased to 50%. These results suggest that the type frequency of regular exemplars indeed needs to be relatively high to ensure productivity, as is assumed across theories. Taking the raw numbers of exemplars instead of the proportions, we find that both the learning success and failure in these studies are as predicted by the TP algorithm (Yang, 2016).

However, the productivity threshold remains unclear, as this was never tested in these studies. To do so, the numbers of exceptions in contrasting training conditions (i.e., predicted learning success vs. failure) must be close to the threshold on both sides. Considering Yang (2016)’s algorithm, the 8 exceptions out of 24 total exemplars in the “failure” condition of Gómez and Lakusta (2004) was closely above the tolerance threshold (θ*_N_* =*N*/ln*N* = 24/ln24 *=* 7.55), but the contrasting ‘success’ condition with 4 exceptions (out of 24) was far below the threshold. In Koulaguina and Shi (2019) the exceptions (*e* = 2) in a ‘success’ condition was far below the threshold (θ*_N_* = 10/ln10 = 4.34), whereas the contrasting “failure” condition with 8 exceptions was far above the threshold (θ*_N_* = 16/ln16 = 5.77). Thus, the exceptions in these studies were not set in a way to be informative of the productivity threshold.

The present study aimed at better understanding rule/pattern learning in infants, by testing precise theoretical predictions. Specifically, we directly tested the threshold of productivity. Furthermore, we asked whether productivity is quantal or gradient. We built on our previously published experiments (Koulaguina and Shi, 2013, 2019), in which generalization success and failure were consistent with TP as well as with other pattern-learning theories, but the numbers of exceptions were not designed to test the productivity threshold. We report two new conditions that used the same implicit learning task and the same stimuli (of the word-order movement patterns) as in our prior studies, but where the numbers of exceptions in training were set close to the TP-threshold. Moreover, our new and previous studies form a continuum of training conditions with gradually increasing exceptions, allowing us to analyze the combined data and address the gradience-quantalness question.

In the remainder of this article that report our study, the term “rule” is used for convenience to mean either abstract symbolic rule or non-abstract pattern, without a bias, as our study is not designed to test the distinction of abstract rule representation vs. analogy-based generalization. The learning shown in our study is compatible with both theoretical assumptions.

## 2. Methods

### 2.1. Participants

Forty-eight non-Russian-learning 14-month-olds completed this experiment (Condition 1: mean age = 462 days; range = 446–483; 11 girls; Condition 2: mean age = 459 days; range: 435–478; 12 girls). The data of another eight infants were excluded from analyses because of fussiness (2), out of the camera view during test trials (1), and lack of interest in the task (5).

### 2.2. Stimuli

Stimuli were those of our previously published studies (Koulaguina and Shi, 2013, 2019), adapted for the distributions of our new training conditions. They were three-word sentences in Russian, 16 used for constructing training exemplars and two for testing exemplars. Given the TP-threshold θ*_N_* =*N*/ln*N* = 16/ln16 = 5.77 for the training, we set 11 rule exemplars and 5 exceptions for Condition 1 (i.e., productive), and 10 rule exemplars and 6 exceptions for Condition 2 (unproductive). In terms of proportion, rule exemplars were above 50% in both conditions.

As in our previous studies, the stimuli were prepared by applying each rule sentences to two word-order movement rules. For Rule 1, Words 1 and 2 were switched (i.e., abc-bac), whereas Words 2 and 3 were switched for Rule 2 (i.e., abc-acb). Exceptions were sentences appearing only in the base form (i.e., abc), with no word-order-shifted version. The two test sentences were each applied to the two word-order rules. Table 1 shows the base sentences that we used to construct our stimuli.

**Table 1:** Sentences used in the two conditions.

	Base abc sentences for the word-order shift rules (either abc→bac or abc→acb; each rule exemplar consisted of a base sentence and its shifted version)	Exception sentences (non-shifted; abc)
Training	*Dozhd’ zalil cherdak.* *Veter gnjot derev’ja.* *Vorona nashla pugovitsy.* *Machty gnutsja lukom.* *Zina gladit plat’e.* *Pojte pesnju druzhno.* *Dimke snilos’ pole.* *Chistim tufli vaksoj.* *Budesh vilkoj kushat’.* *Flagi utrom snjali.* *Veter vybil okna.* (removed in Condition 2)	*Stanut reki polny.* *Otzvuk smekha sladok.* *Seno pahnet volej.* *Skrojut tuchi solntse.* *Obuv’ skinul rezvo.* *Tanets veren bubnu.* (added in Condition 2)
Test	*Vizhu nosik belki.* *Snova milyj vessel.*	

The sentences were recordings of a Russian female speaker from Koulaguina and Shi (2013, 2019). The recording was produced in infant-directed speech style. The speech rate was slow such that a brief pause occurred naturally between words in all sentences. The pauses were helpful cues to word segmentation, allowing our task to focus on testing the learning of word order movement without the complication of word segmentation difficulty in an unknown language.

The recorded stimuli were organized as follows. For Condition-1 training, 11 exemplars in Rule 1 (abc-bac) and five exceptions (in abc) formed the input for one subgroup of infants. The 11 same base sentences in Rule 2 (abc-acb) and the same five exceptions formed the input for another subgroup. For Condition-2 training, one of the 11 rule exemplars of Condition 1 was removed, and we added an exception; thus, the 10 remaining Rule-1 exemplars and six exceptions formed the input for one subgroup, and 10 Rule-2 exemplars and the six exceptions for the other subgroup.

Thus, the distributions of the training input were organized in terms of exemplars. For both conditions, each rule exemplar consisted of a base sentence and its shifted version. For example, the sentences *Dozhd’ zalil cherdak* and *Zalil dozhd’ cherdak* constituted one exemplar of the abc-bac rule. Exceptions were singletons in the abc base order. For example, *Stanut reki polny* was counted as one exception exemplar.

To construct our test stimuli, we applied each of the two new test sentences to Rule 1 (abc-bac) and to Rule 2 (abc-acb).

For Condition-1 training, the average sentence duration (with base and shifted versions measured separately) was 2.88 s (SD = 0.46) for Rule 1, 2.86 s (SD = 0.42) for Rule 2, and 2.48 s (SD = 0.18) for the exceptions. For Condition-2 training, the average sentence duration was 2.91 s (SD = 0.47) for Rule 1, 2.89 s (SD = 0.43) for Rule 2, and 2.43 s (SD = 0.20) for the exceptions. The average sentence duration in the test phase was 2.55 s (SD = 0.09) for Rule 1, and 2.55 s (SD = 0.09) for Rule 2.

Visual stimuli included animations of colorful moving circles and moving blue geometric forms. Between trials, a moving star with the sound of birds singing served as the attention-getter. During the pre-trial and post-trial, electric sound of a bouncing ball was used.

### 2.3. Procedure and apparatus

The experiment used two IAC sound-attenuated rooms. In the training room the child and the parent sat on a sofa before two small TV screens. A box of soft toys was available. The parent wore headphones playing masking music and were asked not to talk. They could play together using the toys. *Habit 2* software was used to present the stimuli and record the child’s looking times (Oakes et al., 2019). In an adjacent room, a researcher blind to the stimuli launched the experiment and coded the child’s looking to the screens. The training stimuli were presented fully in one trial, and repeated in three other trials, regardless of whether the child looked at a screen. The order of exemplars was randomized during each trial, with the restriction that the pair of sentences within each rule exemplar always occurred together (for maintaining the cohesion of a rule exemplar), i.e., the base abc sentence followed immediately by its shifted version. The visual display, i.e., the colorful moving circles for the initial three training trials and the blue geometric forms for the last trial, appeared on both screens simultaneously and were presented together with the speech stimuli.

Immediately following training, the parent and the child moved to the other sound-attenuated room with no toy. The infant sat on the parent’s lap facing a large central screen. The parent wore headphones playing masking music. The researcher, blind to all stimuli, launched the experiment and coded the infant’s looking through a monitor using *Habit 2*. The test trials were infant-controlled, i.e., initiated by the infant’s looking and terminated when she looked away from the screen for at least 2 s. A test trial would also terminate if the maximum trial length was reached in case of no lookaway. The presentation of audiovisual stimuli would stop if a trial was terminated (either by the child’s lookaway or by the maximum trial length). The visual stimuli for every trial of the test phase were the blue geometric forms, which was presented together with speech stimuli.

The inter-stimulus interval was 1,200 ms in-between exemplars, and 700 ms separating a base sentence from its shifted version within a rule exemplar.

### 2.4. Design

Within each condition, infants were randomly assigned to one of the two subgroups, either to Rule 1 with exceptions, or to Rule 2 with exceptions. This design ensured that subgroups hearing different rules (during training) served as each other’s control, such that a particular new test exemplar in one rule that was grammatical for one subgroup would be ungrammatical for the other subgroup, and vice versa.

Infants heard four training trials (total duration 412 s for Condition 1 and 400 s for Condition 2). Each trial contained the 16 exemplars (11 rule cases and 5 exceptions for Condition 1; 10 rule cases and 6 exceptions for Condition 2). Table 2 shows the design. Recall that each rule exemplar consisted of a base sentence (abc order) and its shifted version (e.g., bac), and that each exception exemplar was a singleton sentence without a shifted version. To illustrate, Table 3 shows the detailed training exemplars and test stimuli for one of the subgroups of Condition 1.

**Table 2:** Experimental design.

Training (TP-threshold: *e* = 5.77) (statistical-majority threshold: 50%)	Condition 1: 11 abc→bac exemplars 5 exceptions	Condition 1: 11 abc→acb exemplars 5 exceptions
	Condition 2: 10 abc→bac exemplars 6 exceptions	Condition 2: 10 abc→acb exemplars 6 exceptions
Test (new exemplars)	abc→bac (trained rule) abc→acb (non-trained rule)	abc→acb (trained rule) abc→bac (non-trained rule)

**Table 3:** Stimuli for one subgroup of Condition 1.

	Exemplars for the abc→bac word-order shift rule (each rule exemplar consisted of a base sentence and its shifted version)	Exemplars of exceptions (non-shifted; abc)
Training (11 rule exemplars and 5 exemplars of exceptions)	*Dozhd’ zalil cherdak.* → *Zalil dozhd’ cherdak.* *Veter gnjot derev’ja.* → *Gnjot veter derev’ja.* *Vorona nashla pugovitsy.* → *Nashla vorona pugovitsy.* *Machty gnutsja lukom.* → *Gnutsja machty lukom.* *Zina gladit plat’e.* → *Gladit zina plat’e.* *Pojte pesnju druzhno.* → *Pesnju pojte druzhno.* *Dimke snilos’ pole.* → *Snilos’ dimke pole.* *Chistim tufli vaksoj.* → *Tufli chistim vaksoj.* *Budesh vilkoj kushat’.* → *Vilkoj budesh kushat’.* *Flagi utrom snjali.* → *Utrom flagi snjali.* *Veter vybil okna.* → *Vybil veter okna.*	*Stanut reki polny.* *Otzvuk smekha sladok.* *Seno pahnet volej.* *Skrojut tuchi solntse.* *Obuv’ skinul rezvo.*
Test	*Vizhu nosik belki.* → *Nosik vizhu belki.* (abc-bac) *Snova milyj vessel.* → *Snova vessel milyj.* (abc-acb)	

The test phase started with a pre-trial, followed by two introduction trials, 14 test trials and a post-trial. The pre-trial served to familiarize the child with the equipment. The post-trial marked the end of the experiment. The two introduction trials presented the two new sentences that would be later used in the test trials. One sentence was applied to Rule 1 in one introduction trial, and the second sentence was applied to Rule 2 in the other introduction trial. See the examples in Table 3. Once initiated, the two introduction trials were presented in full (each 6,000 ms) so that the child could hear and encode both the base and shifted version of each sentence. In Conditions 1 and 2, the introduction trials were counterbalanced across babies for the specific sentence in which the rules appeared (half heard the first sentence in Rule 1 and the second sentence in Rule 2; the other half heard the first sentence in Rule 2 and the second in Rule 1) and for the order of the presentation of the rules (half heard Rule 1 in the first trial, and the other half heard Rule 2 in the first trial).

The 14 test trials followed the introduction trials, presenting the same two test sentences in their respective rules in alternate trials, with the same counterbalancings as the introduction trials. For example, if the first sentence in Rule 1 was the first introduction trial, it was also the first test trial. The test trials were fully infant-controlled, initiated and terminated by the child’s looking. The maximum length for each test trial was 21 s if the child looked till the end of the trial, and in this case the rule exemplar in the trial would be repeated and heard for a total of three times. Across all counterbalancing subgroups, each infant received two types of test trials according to the training: the trained rule vs. the non-trained rule.

### 2.5. Predictions

Our study was designed to test distinct theoretical predictions. Rule exemplars in the input of the two conditions were 68.75% vs. 62.5%, both greatly surpassing the statistical majority of 50%. Thus, both should show successful learning if statistical majority determines productivity. However, if the onset of productivity is determined by TP, then Condition 1, but not Condition 2, should show success, as the exceptions were set just below the TP-threshold in the former (*e* = 5), but just above the threshold in the latter (*e* = 6).

## 3. Results

**Figure 1:**
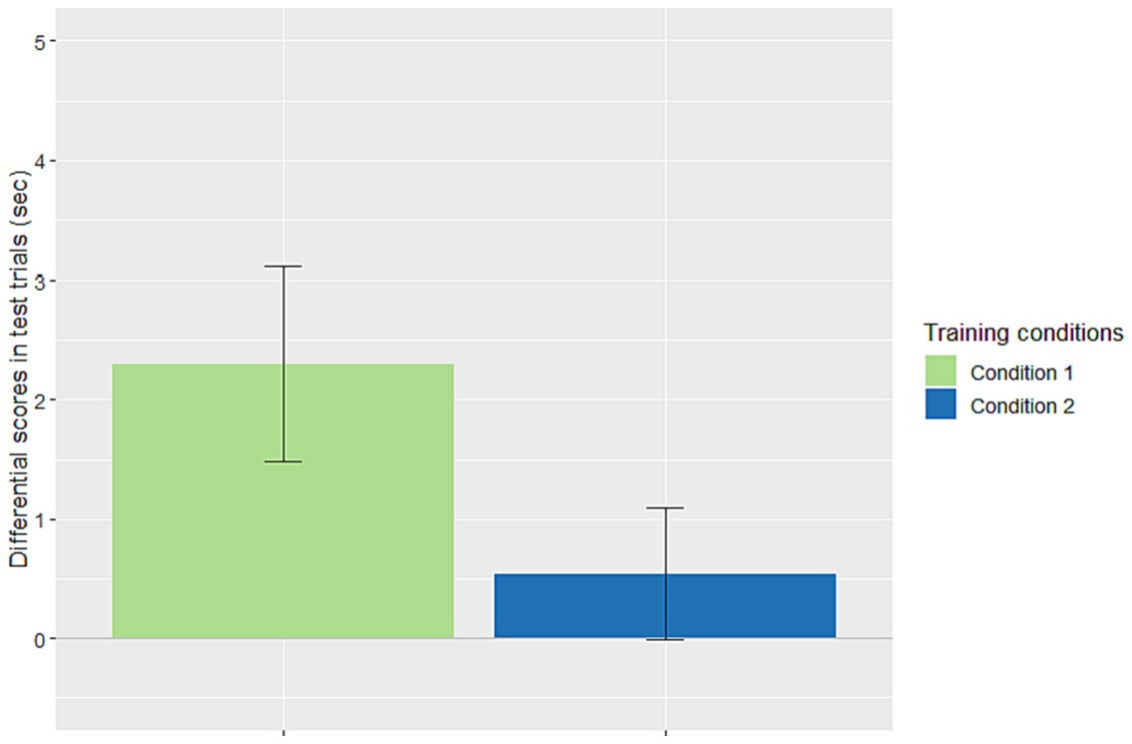
Differential scores (means and standard errors) of looking times in the test trials (i.e., the average looking time per trial for the untrained rule minus that for the trained rule). The differential scores were significantly above chance in Condition 1 [68.75% rule exemplars (11); the number of exceptions *e* = 5 was just below the TP-threshold, θ*_N_* =*N*/ln*N* = 16/ln16 = 5.77], but not in Condition 2 [62.5% rule exemplars (10); the number of exceptions *e* = 6 was just above the TP-threshold 5.77].

We first analyzed whether productivity was present in Conditions 1 and 2. For each infant, the average looking time per trial for each type of test trials (trained rule vs. non-trained rule) was calculated. Then, we calculated the differential score of the looking times for every infant (i.e., non-trained minus trained). Based on the results of our previous studies on rule generalization from the same kind of stimuli in the same task (Koulaguina and Shi, 2013, 2019), we predicted *a priori* that successful generalization should yield a novelty effect (longer looking toward the non-trained rule than the trained rule, i.e., differential scores >0). Specifically, this novelty effect was expected for Condition 1, given that the input distribution should lead to successful generalization according to both statistical majority and TP theories, whereas this may or may not be the outcome of Condition 2 depending on the particular theory. The novelty effect was confirmed for Condition 1, with differential scores significantly above the 0 chance level, *M* = 2.3 s, SE = 0.82, *t*(23) = 2.816, *p =* 0.005. However, the differential scores in Condition 2 did not differ significantly from chance [*M* = 0.54 s, SE = 0.55, *t*(23) = 0.975, *p* = 0.17], suggesting no generalization. These results agree with the predictions of TP (Yang, 2016), but much less with that of statistical majority. We further predicted that the differential scores in Condition 1 should be greater than those in Condition 2, which was confirmed, with respective means being 2.3 s vs. 0.54 s, unpaired *t*(46) =1.785, *p* = 0.04, *Cohen’s d* = 0.515 (see Figure 1). We also conducted a Bayes factor analysis of the data of the two conditions to compare the null hypothesis (i.e., no difference between the conditions) and an alternative hypothesis (the differential scores in Condition 1 greater than those in Condition 2). The analysis was performed with a Cauchy distribution prior for the effect size, a standard choice indicating minimal prior information. We obtained a Bayes factor of 2.067, indicating that the data were two times more likely to occur under the alternative hypothesis than under the null hypothesis. This result was in line with that of the *t*-test.

**Figure 2:**
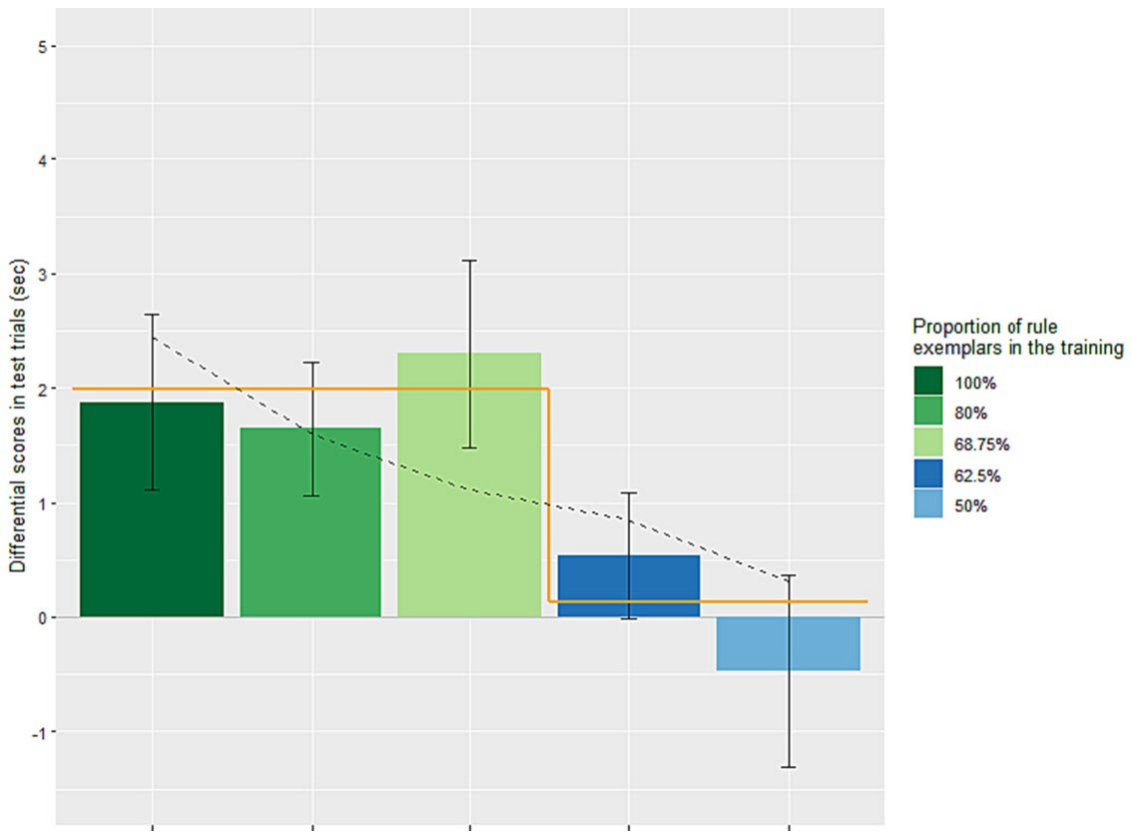
Differential scores (means and standard errors) of looking times of the test trials (i.e., the average looking time per trial for the untrained rule minus that for the trained rule). The five training conditions contained different proportions of rule exemplars relative to exceptions. The 68.75% (i.e., 11R/16) and 62.5% conditions (i.e., 10R/16) were those of the present study. The other conditions with more extreme proportions of rule exemplars were from our previous studies (the 100% condition from Koulaguina and Shi, 2013, and the 80 and 50% conditions from Koulaguina and Shi, 2019), i.e., 10R/10, 8R/10, 8R/16. The 100, 80, and 68.75% conditions showed successful productive learning, and the learning performance across the three conditions was comparable statistically. The 62.5 and 50% conditions showed no productive learning, and their performance was also comparable statistically. The black dashed line across the columns represents the linear regression. Note that the linear regression line is a straight line; however, it has been slightly adjusted due to the uneven spacing between the different proportions of rule exemplars across the five training conditions. The orange line contains two horizontal lines representing the two values of the step function, with the break points between the 68.75 and 62.5% conditions.

Next, we analyzed the data of the present study together with those of our previous studies (see Figure 2), to determine whether productivity is gradient or quantal. Gradient productivity with increasing consistency of regular exemplars is assumed in various models (e.g., Baayen, 1993; Bybee, 1995), whereas TP predicts a quantal shift between non-productivity and productivity at the tolerance threshold. In one previous experiment we presented 50% regular exemplars (*e* = 8, far above the TP-threshold, θ*_N_* =*N*/ln*N* = 16/ln16 = 5.77), and infants showed no discrimination of test trials, i.e., no productivity (Koulaguina and Shi, 2019). Condition 2 of the present study (62.5% regular exemplars), with exceptions (*e* = 6) just above the TP-threshold (5.77), also showed no productivity. The comparison of the differential scores of the previous 50%-experiment (*M* = −0.47 s, SE = 0.84) and those of Condition 2 (62.5% regular exemplars: *M* = 0.54 s, SE = 0.55) showed comparable performance [unpaired *t*(38) = 1.046, *p* = 0.302], that is, no evidence of a gradient difference between these two levels of regularity. Next, we took the data from two previous experiments that showed rule productivity, one presenting 100% regular exemplars (Koulaguina and Shi, 2013), and another 80% regular exemplars with exceptions (*e* = 2) far below the TP-threshold (θ*_N_* =*N*/ln*N* = 10/ln10 = 4.34) (Koulaguina and Shi, 2019). We calculated the differential scores of test trials (i.e., non-trained minus trained) of each infant in those experiments (100% regular exemplars: *M* = 1.87 s, SE = 0.77; 80% regular exemplars: *M =* 1.64 s, SE = 0.58), and analyzed them together with the differential scores of Condition 1 of the present study [68.75% regular exemplars with exceptions (*e* = 5) just below the TP-threshold 5.77] in a one-way ANOVA. The scores did not differ across the three conditions [*F*(2, 53) = 0.198, *p* = 0.821], indicating no evidence of gradually decreasing performance, as was already visible in the pattern of their means (1.87, 1.64 and 2.3). Taken together, the lack of difference in the above two statistical comparisons suggest that the learning was not gradient.

We conducted additional analyses of the five experiments to further assess if infants’ generalization was gradient-like or quantal-like. First, a linear regression analysis was done, using the differential score as the dependent measure and the five levels of training as the independent measure. The results revealed that the levels of training significantly predicted the differential scores, *F*(1, 94) = 4.16, *p* = 0.0442. The regression model explained a small proportion of the variance in differential scores (adjusted *R*^2^ = 0.032). Notably, each increase of 1% in regular exemplars in training was associated with a positive change in the differential score, with a coefficient of *B* = 0.043 (β = 0.206). This positive trend was compatible with both gradient and quantal performance.

Therefore, we subsequently assessed if infants’ performance showed a discrete change from 62.5 to 68.75% training. A step function model compared the TP-predicted unlearnable conditions, scored 0, and the TP-predicted learnable conditions, scored 1, in the format of a regression. This approach allowed us to compare the adjusted R^2^ of the step function model with that of the initial linear regression based on the five levels of training as the predictor. The results of the step function analysis indicated that the binary learnability index significantly predicted the differential scores, *F*(1, 94) = 8.00, *p* = 0.0057. The step function model explained still a small proportion of the variance in differential scores, although more than twice than was explained by the gradually increasing model (adjusted *R*^2^ = 0.068 in the step function, compared to 0.032).

When the predictors based on the above two analyses (i.e., the gradual and the single step increase functions) were entered together, the overall fit degraded [*F*(1, 93) = 3.96, *p* = 0.0224, adjusted *R*^2^ = 0.059] relative to the second analysis alone, due to the loss of one degree of freedom. Importantly, the test for the step function, once the other predictor is taken into account, still approached significance, despite the loss of one degree of freedom [*t*(93) = 1.91, *p* = 0.0595], while that for the gradual increase predictor, after taking the other predictor into account, indicated a total absence of effect [*t*(93) = 0.002, *p* =0.9985]. Overall, the results of the above analyses were consistent with quantal rather than gradient performance.

Finally, we analyzed if infants received the same amount of active exposure during training, measured by their looking time to the screens, where the sound source was. Results showed no difference in looking during training in Condition 1 (*M* = 110.73 s, range = 33.23–273.77) vs. Condition 2 (*M* = 112.09 s, range = 31.62–289.44), *t*(46) = −0.07, *p* = 0.944. Individual infants’ looking times during training did not correlate with their differential scores in the test, neither in Condition 1 (*r* = −0.135, *n* = 24, *p* = 0.53) nor Condition 2 (*r* = 0.008, *n* = 24, *p* = 0.972). Infants spent more time playing with the toys and with the parent. Their looking times were low (on average about 1/4 of the training duration) and variable. However, infants in each subgroup received the same full passive exposure of the training input, suggesting that their performance during test reflected implicit/unconscious learning.

## 4. Discussion

We examined infants’ generalization from input containing different levels of regular exemplars and exceptions. Globally, productivity depended on regular exemplars being high in type frequency relative to exceptions, as predicted in all rule/pattern learning theories (e.g., Baayen, 1993; Bybee, 1995; Yang, 2016). Most theories focus on ranking different patterns with varying productivity indices, without specifying a threshold of productivity. TP (Yang, 2016), in contrast, specifies an algorithm for the threshold of learning any rule, calculated from the number of rule exemplars and exceptions. Our training input was therefore set closed to the TP-threshold, with Condition 1 predicting productivity and Condition 2 no productivity. In terms of proportions, the regular exemplars were in statistically majority (over 50%) in both conditions (68.75, 62.5%), contrasting with the 50% training in one of our previous experiments (which yielded no learning) (Koulaguina and Shi, 2019). If statistical majority is the productivity threshold, infants should show generalization in both Conditions 1 and 2 of the present study. Therefore, statistical majority was pitted against Yang’s TP-threshold. For these three training conditions (68.75, 62.5, 50%), the number of total types was equal (*N* = 16) whereas the regular exemplars vs. exceptions varied (11 + 5, 10 + 6, 8 + 8). Infants showed productivity only in the 11 + 5 (68.75%) condition, consistent with TP, but not with statistical majority.

Another novel finding emerged from the combined analysis of our present and previous experiments: Infants’ productivity was quantal rather than gradient. As existing theories are often concerned with ranking the levels of productivity of regulars and irregulars within a rule/pattern or even unrelated rules/patterns (e.g., comparing English affixal rules such as *re*-, -*able*, -*ness*), their assumption is that productivity levels are gradient (e.g., Baayen, 1993; Bybee, 1995). In contrast, according to TP, productivity is quantal, depending on whether the exceptions to a rule sit below or above the TP-threshold (Yang, 2016); a productive rule, once learned, is equally productive regardless of the number of exceptions. Our infants showed evidence not only for the TP-predicted threshold, but also for the quantal effect. Infants in different learnable conditions were equally successful, whether input consistency was 100, 80%, or 68.75%. Likewise, the conditions that yielded no learning did not differ from each other. The linear regression and step function analyses indicated that the learning was categorical, rather than gradient. That is, infants responded quantally, as did older children in Bjornsdottir (2021). The lack of productivity gradience in infants is consistent with the regularization performance (rather than probability matching) shown in 5- to 7-year-old children (Hudson Kam and Newport, 2005, 2009; Austin et al., 2022).

Finally, we showed that rule/pattern learning can be implicit. In other studies testing TP with older children and adults (Schuler et al., 2016; Bjornsdottir, 2021), training required participants to maintain active attention. In our present and previous experiments (Koulaguina and Shi, 2013, 2019) infants relied mostly on passive input exposure, suggesting that learning can also operate at an unconscious neurocognitive level, at least for certain kinds of knowledge. Similarly, in Saffran et al. (1997) adults and 6- to 7-year-olds passively tracked transitional probabilities in an artificial language while actively performing another task unrelated to the background training speech. Since infants and toddlers, who typically have limited attention span, receive abundant passive/incidental exposure to various stimuli in daily life (e.g., language input), the implicit learning demonstrated by our infants offers insight into mechanisms of early cognitive and language development.

Exceptions used in TP studies are typically overt violation cases (comparable to the irregular past-tense form *went* in English). The exceptions in our input, however, are not direct violations of a rule/pattern but absence of evidence, a scenario occurring commonly in natural speech and important for language learnability theories. Our results show that such cases function equivalently as overt violation cases for learners; for both kinds, it is the number of positive data (i.e., attested regular cases) that determines productivity.

In conclusion, our findings suggest that rules or patterns can be learned from inconsistent input implicitly without attention. The evidence supports the threshold and quantalness of productivity as defined in TP (Yang, 2016). We demonstrate both success and failure of rule/pattern learning and generalization as predicted by the theory in infants as young as 14 months of age, indicating that the mechanism is available early in life.

## Data availability statement

The datasets presented in this study can be found in online repositories. The names of the repository/repositories and accession number(s) can be found at: https://osf.io/pe62r/.

## Ethics statement

The studies involving humans were approved by Comité institutionnel d’éthique de la recherche avec des êtres humains (CIEREH), Université du Québec à Montréal. The studies were conducted in accordance with the local legislation and institutional requirements. Written informed consent for participation in this study was provided by the participants’ legal guardians/next of kin.

## Author contributions

RS designed the study, guided the construction of the experiment and the data analysis, and wrote the article. EE prepared the experiment, tested the participants, and analyzed the data. All authors contributed to the article and approved the submitted version.

## Footnotes

^1^Hare et al. (1995) further showed that in the case of a low-type-frequency default inflection alongside other non-default inflections, their models succeeded in generalizing the default pattern. Whereas a default inflection had no specified phonological template for the stem, the stems of the non-default inflections had well-defined phonological shapes. Their models learned the non-default inflections as different processes irrelevant to the default process, rather than treating them as exceptions to the default. In this sense, the non-default processes were not in competition with the default; as a result, the type frequency of the default regular exemplars was still dominant relative to true exceptions (i.e., those that did not comply with the default pattern and also did not resemble the stem templates of the non-defaults), and thus the default was generalizable.
